# Altered Functional Connectivity during Mild Transient Respiratory Impairment Induced by a Resistive Load

**DOI:** 10.3390/jcm13092556

**Published:** 2024-04-26

**Authors:** Akiko Yorita, Tomotaka Kawayama, Masayuki Inoue, Takashi Kinoshita, Hanako Oda, Yoshihisa Tokunaga, Takahisa Tateishi, Yoshihisa Shoji, Naohisa Uchimura, Toshi Abe, Tomoaki Hoshino, Takayuki Taniwaki

**Affiliations:** 1 Division of Respirology, Neurology, and Rheumatology, Department of Medicine, Kurume University School of Medicine, Kurume 830-0011, Japan; akiyori519610@yahoo.co.jp (A.Y.); kawayama_tomotaka@med.kurume-u.ac.jp (T.K.); tkino@med.kurume-u.ac.jp (T.K.); odahana@med.kurume-u.ac.jp (H.O.); tokuyoshi0622@kurume-u.ac.jp (Y.T.); tateishi_takahisa@med.kurume-u.ac.jp (T.T.); hoshino@med.kurume-u.ac.jp (T.H.); 2 Cognitive and Molecular Research Institute of Brain Disease, Kurume University, Kurume 830-0011, Japan; inouemas64@gmail.com (M.I.); yshoji@med.kurume-u.ac.jp (Y.S.); naohisa@med.kurume-uu.ac.jp (N.U.); 3 Department of Radiology, Kurume University School of Medicine, Kurume 830-0011, Japan; toshiabe@med.kurume-u.ac.jp

**Keywords:** resting state, functional connectivity, transient respiratory impairment, resistive load, effort breathing

## Abstract

**Background**: Previous neuroimaging studies have identified brain regions related to respiratory motor control and perception. However, little is known about the resting-state functional connectivity (FC) associated with respiratory impairment. We aimed to determine the FC involved in mild respiratory impairment without altering transcutaneous oxygen saturation. **Methods**: We obtained resting-state functional magnetic resonance imaging data from 36 healthy volunteers during normal respiration and mild respiratory impairment induced by resistive load (effort breathing). ROI-to-ROI and seed-to-voxel analyses were performed using Statistical Parametric Mapping 12 and the CONN toolbox. **Results**: Compared to normal respiration, effort breathing activated FCs within and between the sensory perceptual area (postcentral gyrus, anterior insular cortex (AInsula), and anterior cingulate cortex) and visual cortex (the visual occipital, occipital pole (OP), and occipital fusiform gyrus). Graph theoretical analysis showed strong centrality in the visual cortex. A significant positive correlation was observed between the dyspnoea score (modified Borg scale) and FC between the left AInsula and right OP. **Conclusions**: These results suggested that the FCs within the respiratory sensory area via the network hub may be neural mechanisms underlying effort breathing and modified Borg scale scores. These findings may provide new insights into the visual networks that contribute to mild respiratory impairments.

## 1. Introduction

The recognition of breathing and the breathing impairment (dyspnoea) are important not only in respiratory and cardiac diseases but also in neurological disorders such as amyotrophic lateral sclerosis and myasthenia gravis. Respiratory centres in the brainstem regulate automatic respiratory rhythms [1], but brain regions associated with respiratory motor control and breath perception have only recently been described [2]. Recent advances in neuroimaging have provided new insights into functional impairments in the human brain [3]. Several studies using a box-car design, task-based positron emission tomography (PET), and conventional functional magnetic resonance imaging (fMRI) have identified brain regions associated with respiratory motor control and perception of dyspnoea [2].

fMRI research has shifted its focus from identifying regions specialised for cognitive tasks (conventional fMRI) to understanding broader interactions between multiple brain regions. This concept is known as functional connectivity (FC), which refers to synchronisation of neural activity between regions [4]. Using resting-state fMRI techniques, a growing body of research has revealed altered FC in brain disorders such as Alzheimer’s disease [5], depression [6], and schizophrenia [7]. FC has been studied in respiratory disorders such as obstructive sleep apnoea [8] and chronic obstructive pulmonary disease [9], but these findings may be due to prolonged hypoxia and hypercapnia. A network-based approach to task-related FC linked specific areas (the anterior insular (AInsula) and pre-supplementary motor area (SMA)) to tasks involving breathing restriction and craving regulation [10]. Consequently, our comprehension of altered FC during acute respiratory impairment remains limited.

Analysing FC requires 100–300 whole-brain volumes and an acquisition time of 5–10 min [11]. Previous respiratory-focused neuroimaging studies often used tasks such as breath-holding, which were difficult to sustain for the required data acquisition period [12,13,14]. To address this, we designed a mouthpiece device that resists breathing flow through the mouth, enabling investigations lasting over 5 min [15]. This allows us to examine the association between FC and mild dyspnoea induced by a resistive load (effort breathing) using resting state fMRI.

Conventional fMRI studies have reported that effort breathing affects the motor and sensory processes associated with dyspnoea, including the sensory-motor cortex, premotor cortex, SMA, insular cortex, anterior cingulate cortex (ACC), amygdala, thalamus, basal ganglia, cerebellar hemisphere, cerebellar vermis, and brainstem (midbrain, pons, and medulla) regions [2,16,17,18,19,20,21].

In this study, we employed whole-brain ROI-to-ROI analysis to screen for changes in FC during mild dyspnoea as well as further examine these changes at the voxel-level using seed-to-voxel analysis. Specially, we aimed to (1) investigate the FC associated with the processing of mild dyspnoea caused by forced breathing; (2) compare the regions identified by conventional fMRI studies [16,17,18,19,20,21]; and (3) explore the relationship between clinical scores. We hypothesised that effort breathing will activate FCs involving previously reported brain regions [16,17,18,19,20,21].

## 2. Materials and Methods

### 2.1. Participants

The study enrolled never-smokers aged 20–50 years with stable health, normal lung function, no respiratory symptoms, and no medication use. Exclusion criteria included a history of lung surgery, severe diseases of other organs, pregnancy or nursing status, dementia, or drug or alcohol abuse. 

### 2.2. Induction and Measurement of Respiratory Impairment

The artificial dyspnoea was performed using a cylindrical polyethylene device (24 mm inner diameter, 26 mm outer diameter, and 900 mm length), with a mouthpiece at one full-opened end with 6.3% of corresponding aperture ratio opened 6.0 mm-diameter sizes at the opposite side (patent application no. 2010-160596, Fukuda Denshi, Tokyo, Japan) (Figure S1) [15]. Our previous study [15] demonstrated that, compared with the mouthpiece alone, the device significantly increased the mean peak mouth pressure (mouthpiece vs. device: 0.01 to 0.00 kPa/L/s vs. 0.02 to −0.02 kPa/L/s) as measured by the flow-pressure meter (SP-370COPDhaiPer; Fukuda Denshi, Tokyo, Japan), and the mean respiratory system resistance (0.25 hPa/L/s vs. 1.04 hPa/L/s) at 5 Hz during quiet breathing for at least 30 s, as determined by the forced oscillation technique (MasterScreen IOS, Jaeger GmbH, Heinsberg, Germany). Additionally, the mean modified Borg scale with the device (3.7) was significantly higher than that with the mouthpiece alone (1.0; resting state) (*p* < 0.0001) [15]. Dyspnoea levels were assessed using the self-reported modified Borg scale for each participant [22,23].

### 2.3. Experimental Design

**Figure 1:**
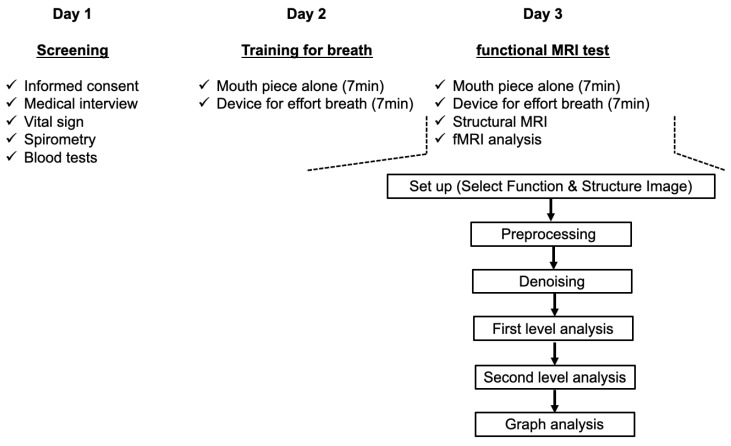
Experimental procedure.

Data were collected during three separate visits (Figure 1). On the first day, participants provided written informed consent and underwent a medical history assessment. The participants then underwent vital sign measurements, including cognitive levels using the modified Hasegawa dementia scale [24], respiratory rate, heart rate, blood pressure, body temperature, modified Borg scale score, and resting SpO_2_. Spirometry and blood tests were also performed. Enrolment criteria encompassed meeting the following thresholds: 30 points on the modified Hasegawa dementia scale, respiratory rates < 20 breaths/min, systolic blood pressure < 150 mmHg, heart rate 40–80 beats/min, body temperature < 37.0 °C, modified Borg scale < 0.5, SpO_2_ > 96%, and normal lung function (% forced vital capacity (%FVC) prediction > 80%, % forced expiratory volume in 1 s (%FEV_1.0_) prediction > 80% of prediction, and the FEV_1.0_/FVC ratio > 0.7). On the second day, vital signs, SpO_2_, and the modified Borg scale [22,23] were monitored every minute over a 14 min period. The first 7 min involved breathing through the mouthpiece alone, followed by 7 min of continuous breathing using a dyspnoea-inducing device. The average modified Borg scale scores were calculated over 1 min intervals during both device-assisted and mouthpiece breathing. Given the challenge of measuring the Borg scale in the MRI scanner and its repeatability within 7 days, we considered the data from day 2 to be equivalent to those from day 3. Within 7 days of day 2, fMRI was conducted under identical conditions to the breathing training on day 2 (day 3). During the fMRI tests, each participant had two consecutive imaging series over 400 s for both respiratory conditions, one for normal respiration (mouthpiece only) and one for mild dyspnoea (device with mouthpiece). The order of normal and effort breathing was counterbalanced among the participants. Vital signs, modified Borg scale values, and SpO_2_ were compared between the normal and effort breathing conditions on each time course using Student’s *t*-tests, and between baseline and subsequent time points, one-way analysis of variance (ANOVA) with the nonparametric Kruskal–Wallis test (day 2). The average modified Borg scale score during the 7 min training period was correlated with the fMRI data and immediately re-evaluated after the fMRI.

### 2.4. MRI Data Acquisition

Images were acquired using a 3T GE Discover MR 750 W whole-body MRI system. Each session consisted of acquiring 200 echo-planner imaging multi-slice datasets (repetition time, 2000 ms; echo time, 30 ms; flip angle, 80°; acquisition time, 400 s) while the participants were instructed to relax with their eyes closed. Each multi-slice dataset comprised 34 transverse slices (slice thickness, 4 mm; voxel size, 3.5 × 3.5 × 4 mm^3^; field of view (FOV), 220 mm). High-resolution T1-weighted structural brain images were obtained using a 3D SPGR protocol (repetition time, 8.472 ms; echo time, 3.268 ms; inversion time, 45 ms; FOV, 240 mm; acquisition matrix, 512 × 512; 144 contiguous axial slices; slice thickness, 1.2 mm; and total scan time, 4 min, 3 s). 

### 2.5. Data Pre-Processing

The CONN FC toolbox (version 22a) (https://www.nitrc.org/projects/conn, accessed on 25 March 2023, NITRC, NIBIB, NIH, Bethesda, Rockville, MD, USA) [25], coupled with Statistical Parametric Mapping (SPM 12, Wellcome Department of Cognitive Neurology, University Collage, London, UK) running under Matlab Release 2022b (The MathWorks, Inc., Natick, MA, USA), was used to execute the CONN default pre-processing pipeline as previously described [26,27,28]. This includes the removal of initial functional scans (10 scans); functional realignment and unwarp (subject motion estimation and correction); functional centring to (0, 0, 0) coordinates (translation); functional slice-timing correction; functional outlier detection (identifying outlier scans exceeding the standard deviation (SD) of the average signal or >0.5 mm frame-wise displacement using the ART toolbox); functional direct segmentation and normalisation (simultaneous grey/white/cerebrospinal fluid (CSF) segmentation and Montreal Neurological Institute (MNI) normalisation); structural centring to (0, 0, 0) coordinates (translation); structural segmentation and normalisation (simultaneous grey/white/CSF segmentation and MNI normalisation); and functional smoothing (8 mm full-width half maximum Gaussian kernel filter). Subsequently, denoising steps were implemented, involving CompCor and bandpass filtering in the range of 0.008–0.09 Hz [26,27,28]. To ensure data quality, an exclusion criterion was set with a threshold of 30% for invalid/outlier scans detected by ART.

### 2.6. ROI-to-ROI Analysis

For the first-level ROI-to-ROI analysis, whole-brain ROIs (164 ROIs) from the Harvard–Oxford Atlas [29,30,31,32] and cerebellar ROIs from the Automated Anatomical Labelling Atlas were utilised [33]. Both atlases were integrated into the CONN toolbox [25]. For each ROI, the mean resting-state BOLD time course was extracted to generate individual correlation maps encompassing the entire brain. Correlation coefficients were computed between each ROI’s BOLD time course and those of other ROIs. Fisher’s transformation converts these coefficients into normally distributed scores, facilitating a second-level general linear model analysis [25]. FC measures were computed between the seed regions in the ROI-to-ROI analysis, revealing ROI-to-ROI connectivity patterns through bivariate correlations [25].

Second-level ROI-to-ROI analyses used paired *t*-tests to compare connectivity differences between the normal respiration (mouthpiece alone) and effort-breathing (plus a cylindrical device) groups using paired *t*-tests. Cluster-level analysis, accessible through the CONN toolbox (https://web.conn-toolbox.org/fmri-methods/cluster-level-inferences, accessed on 18 March 2024), was employed to evaluate inter-condition disparities using parametric statistics based on functional network connectivity [27,28,34]. Comparisons were performed using a cluster level threshold with a false discovery rate (FDR) corrected for *p* < 0.05, and connection level threshold at an uncorrected *p* < 0.001 [27].

### 2.7. Seed-to-Voxel Analysis

Seeds for seed-to-voxel analysis were determined from regions that were also reported in previous studies [16,17,18,19,20] among the present ROI-to-ROI analysis results, using CONN’s default atlas for the definition of ROIs referred to as the Harvard–Oxford atlas [29,30,31,32]. The mean BOLD time series from these seed regions was extracted and correlated with the time course of each voxel of the brain, resulting in a three-dimensional correlation coefficient (*r*) map for each subject and seed. Normalised Fisher-transformed correlation maps were used for group analysis (normal respiration vs. breathing effort). In the second-level analysis of Conn’s pipeline, differences in connectivity between normal respiration and effort breathing were measured using paired *t*-rests. For each seed ROI, voxel-wise statistics throughout the brain were calculated. The voxel-level threshold was a *p*-uncorrected value of <0.001, which was then corrected *p* < 0.05 for clusters of voxels by FDR [28]. 

### 2.8. Graph Theoretical Analysis

To identify network hubs, we conducted a graph theoretical analysis using the CONN-fMRI Toolbox. This required considering measures of network centrality, focusing on degree centrality and betweenness centrality. The degree of a node is the number of connections it has with other nodes, whereas the betweenness centrality of a node is the number of times that a node is included in the shortest path from each node to all other nodes [35,36]. The node included all 164 ROIs, and the threshold of the network edge (adjacency matrix threshold) was set to 0.25 in the correlation coefficient (*r*). Within the regions detected by ROI-to-ROI with cluster analysis, a significantly weighted degree and betweenness centrality were identified (uncorrected, *p* < 0.05). We also measured the rank of centrality among all ROIs.

### 2.9. Correlation with the Modified Borg Scale

To determine correlations with the modified Borg scale, we extracted ROIs in which significant group differences were found between effort and normal respiration in the ROI-to-ROI analysis. For each participant, the mean FC values across all voxels in each ROI were computed using seed-to-voxel analysis. Spearman’s rank correlation analysis was conducted to evaluate the relationship between the z-scores of the identified FCs and the modified Borg scales using SPSS (version 26; IBM Corp., Armonk, NY, USA). The statistical significance level was *p* < 0.05.

## 3. Results

### 3.1. Participant Selection

**Figure 2:**
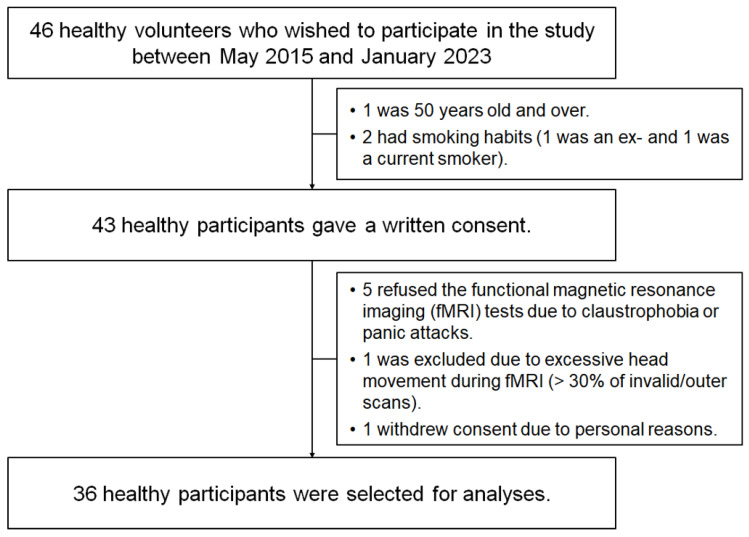
Participant selection.

Forty-six healthy volunteers participated in the study between May 2015 and January 2023. Our selection criteria included individuals aged 20–50 years who had never smoked (see Methods and Participants). Candidates aged >50 years (*n* = 1) and current or former smokers (*n* = 2) were excluded. Furthermore, seven applicants were excluded owing to MRI refusal due to claustrophobia or panic attack (*n* = 5), excessive head movement during fMRI (>30% of invalid/outlier scans detected by Artefact Detection Tools (ART)) (*n* = 1), and the withdrawal of consent for personal reasons (*n* = 1). The remaining 36 participants (18 women and 18 men) were selected for further analysis (Figure 2, Table 1). Notably, all participants reported dyspnoea levels of 0 at rest on the modified Borg scale and scored 30 points on the revised Hasegawa dementia scale [24]. None of the participants showed abnormal pathological findings on anatomical T1- or T2-weighted MRI.

### 3.2. Changes in the Modified Borg Scale and Vital Signs during Respiratory Training between Normal Breathing and Effortful Breathing 

Figure S2 illustrates the time course of the modified Borg scale [22,23], percutaneous oxygen saturation (SpO_2_, %), respiratory rate (breaths/min), blood pressure (mmHg), and heart rate (beats/min). After the MRI, the re-evaluated mean modified Borg scale with effort breathing (0.90 ± 0.84, *p* < 0.0001 using a non-paired *t*-test) was significantly higher than that of normal breathing (0.21 ± 0.44). Notably, no significant differences in SpO_2_ and respiratory rates were observed between effort breathing and normal respiration across time courses or between baseline and subsequent time courses.

### 3.3. Adverse Events

Four participants experienced mild adverse events. All mild adverse events occurred during respiratory training and resolved after training. Transient adverse effects were observed, including tachycardia (*n* = 1), transient hypopnoea (*n* = 1), and transient hypertension (*n* = 1), using the device. Oxygen saturation dropped from 97% to 94% a minute after using the mouthpiece alone but recovered to 95% a minute later. None of the participants experienced any subjective symptoms.

### 3.4. ROI-to-ROI Results

**Figure 3:**
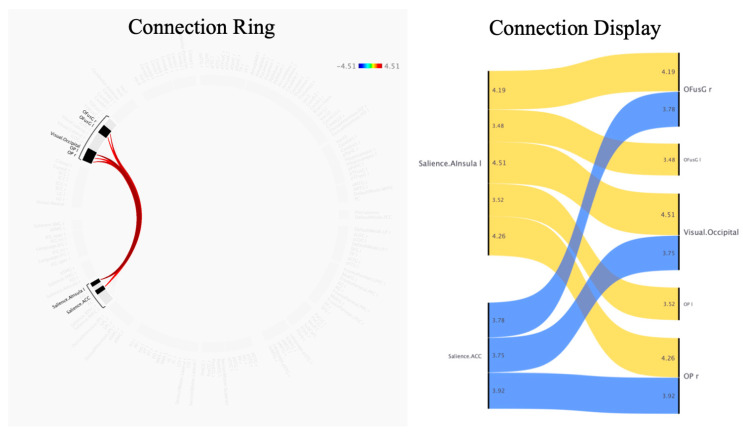
Differences in functional connectivity between effort breathing and normal respiration (effort breathing > normal respiration) are represented by ROI-to-ROI analysis. Colours and numerals represent the *t*-value. Abbreviations: l, left; r, right; ACC, networks’ salience anterior cingulate cortex; AInsula, networks’ salience anterior insular; OFusG, occipital fusiform gyrus; OP, occipital pole.

Compared with normal respiration, effort breathing activated a cluster of FCs between the salience network (including the ACC, and left anterior insular (AInsula)) and a secondary visual network (including visual occipital (VO), bilateral occipital pole (OP), and right occipital fusiform gyrus (OFusG)) (F (3, 68) = 9.16, *p-FDR* = 0.011618) including 8 positive connectivity changes (between the left salience (Sal) AInsula and VO, *t* = 4.51, *p*-FDR = 0.004139; between the left Sal AInsula and right OP, *t* = 4.26, *p*-FDR = 0.004326; between the left Sal AInsula and right OFusG, *t* = 4.19, *p*-FDR = 0.004326; between the Sal ACC and right OP, *t* = 3.92, *p*-FDR = 0.019694; between the Sal ACC and right OFusG, *t* = 3.78, *p*-FDR = 0.019694; between the Sal ACC and VO, *t* = 3.75, *p*-FDR = 0.019608; between the left Sal AInsula and left OP, *t* = 3.52, *p*-FDR = 0.028116; between the left Sal AInsula and left OFusG, *t* = 3.48, *p*-FDR = 0.028116) (Figure 3, Table 2). Only the ACC and left AInsula belong to the regions reported in prior studies [16,17,18,19,20,21]; therefore, both were selected as seeds for subsequent analysis. 

### 3.5. Seed-to-Voxel Results 

**Figure 4:**
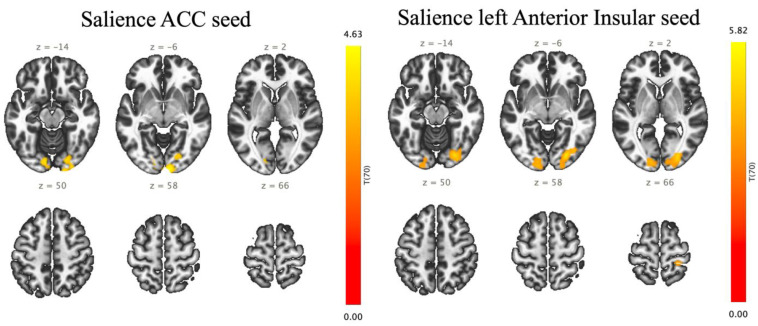
Altered functional connectivity induced by effortful breathing compared with normal respiration (effort breathing > normal respiration) using seed-to-voxel analysis: seed regions are presented above. Colours and numerals represent the positive t-value in yellow/red.

The seed-to-voxel analysis represents that effort breathing increased FCs between the salience ACC and the right OP (303 voxels (35%) covering 12% of OP r) and right OFusG (194 voxels (23%) covering 22% of OFusG r) (size of contiguous voxels = 858; *p-FDR* = 0.000018) (Figure 4, Table 3). Effort breathing significantly activated FCs between the seeds (the left A-Insula) with three different clusters, named A, B, and C. In cluster A (size of contiguous voxels = 1145; *p-FDR* = 0.000001), the left AInsula showed increased connectivity with the right OP (349 voxels (30%) covering 14% of OP r) and right OFusG (317 voxels (28%) covering 36% of OFusG r); in cluster B (size of contiguous voxels = 572; *p-FDR* = 0.000291), the left AInsula disclosed increased connectivity with the left OP (329 voxels (58%) covering 12% of OP l); in cluster C (size of contiguous voxels = 219; *p-FDR* = 0.031177), the left AInsula represents increased connectivity with the right postcentral gyrus (PostCG) (185 voxels (84%) covering 6% of PostCG r). 

### 3.6. Measurement of Nodal Centrality

**Figure 5:**
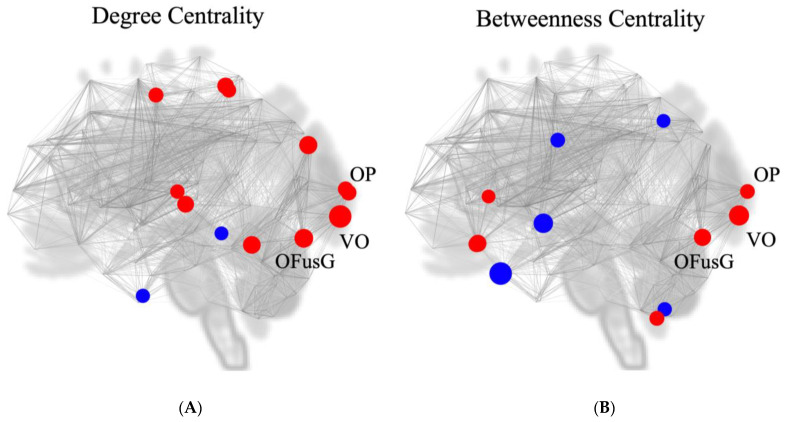
Location of putative hubs in the comparison between effortful breathing and normal respiration (effort breathing > normal respiration). (**A**) Degree centrality (two-sided); (**B**) betweenness centrality (two-sided). Abbreviation: l, left; r, right; OFusG, occipital fusiform gyrus; OP, occipital pole; VO, network’s visual occipital. Red circle shows positive centrality, while the blue circle reveals a negative one.

To investigate the potential of the visual cortex as a network hub, we performed two measures of nodal importance (degree and betweenness centrality) using graph theoretical analysis. During effort breathing, significant values of degree centrality were observed at the visual networks (VO, *t* = 3.26, *p* = 0.001706, first place in all ROIs; left OFusG, *t* = 2.71, *p* = 0.008518, second place; left OP, *t* = 2.26, *p* = 0.026699, seventh place; right OP, *t* = 2.20, *p* = 0.031157, eighth place) and in-betweenness centrality (VO, *t* = 2.93, *p* = 0.004589, first place in all ROIs; left OFusG, *t* = 2.49, *p* = 0.015310, fifth place; left OP, *t* = 2.14, *p* = 0.035570, eighth place) (Figure 5, Table 4).

### 3.7. Correlation with the Modified Borg Scale 

**Figure 6:**
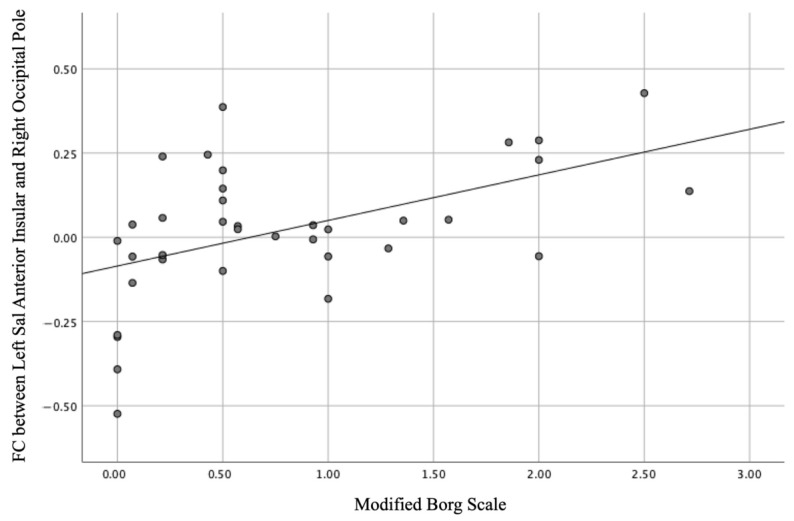
Linear correlation of the modified Borg scale with the functional connectivity between the left anterior insular and right occipital poles (*r* = 0.489, *p* = 0.002).

Eight regions were tested against the modified Borg scale based on networks that showed significant differences between groups in our ROI-to-ROI analysis. Strong positive correlations were observed between the mean scores of the modified Borg scale and the standardised intensity of the FC between the left AInsula and right OP (*r* = 0.489, *p* = 0.002) in the group with effortful breathing (Figure 6).

## 4. Discussion

In this study, we used cluster-level ROI-to-ROI, seed-to-voxel, and graph theory analysis to examine changes in FC associated with transient mild effort breathing. Our novel findings can be summarised as follows: (1) We successfully induced mild respiratory impairment for nearly 7 min in all participants with no obvious changes in SpO_2_. (2) In ROI-to-ROI analysis, effort breathing enhances FCs between the salience network and the secondary visual network. (3) Seed-to-voxel analysis revealed changes in FC between the left AInsula and right postCG. (4) Graph theory analysis showed significant nodal centrality within the visual network. (5) A significant positive relationship was found between the FC (between the left AInsula and right OP) and the clinical score (modified Borg scale). 

### 4.1. Unique Point of Our Resistive Load

Mild respiratory impairment was induced for approximately 7 min. Recent research has highlighted that moderate breathlessness (modified Borg scale score exceeding 3) affected SpO_2_ [37] and oxy-haemoglobin concentration [38]. Furthermore, the potential impact of moderate-to-severe dyspnoea on blood oxygen level-dependent (BOLD) contrast, a core element of fMRI [39], highlights the significance of understanding its influence. Notably, our utilisation of a “grade II” cylindrical device to induce mild dyspnoea without affecting SpO_2_ represents a unique aspect of our study.

### 4.2. Functional Connectivity within the Respiratory-Associated Area

In our ROI-to-ROI and seed-to-voxel analyses, as expected, effort breathing increased FCs between the salience networks (ACC and AIusula) and secondary visual networks (VO, OP, and OFusG) compared with normal respiration. The ACC is interconnected with the primary sensory cortex [40] and plays an important role in regulating autonomic, visceromotor, and endocrine functions associated with emotion [41]. Meanwhile, the insular cortex helps in the perception of various unpleasant sensations, such as pain [42], hunger [43], and negative emotions [44]. AInsular is typically associated with social and affective tasks involving pain, empathy, disgust, and introspective processes [45,46], whereas ACC is closely associated with response selection, conflict resolution, and cognitive control [47]. Together, they shape the salience network’s role in integrating sensory, emotional, and cognitive information [45,48]. Furthermore, anatomical, and electrophysiological studies have demonstrated reciprocal connections between insular and medullary respiratory neurons [49]. Our seed-to-voxel analysis also found increased FC between the left AInsular and right PostCG. Previous reports focusing on acute models of respiratory impairment showed that ACC, AInsula, and PostCG are activated during classic fMRI investigations of the respiratory sensory regions (employing methods such as hypercapnia and air hunger) [2,50,51]. 

### 4.3. Functional Connectivity Outside of the Respiratory-Associated Area

We observed an enhancement of FC within the secondary visual cortex, including the VO, OP, and OFusG, with effort breathing. Conventional fMRI studies on respiratory, sensory, and motor processing have reported no involvement of the visual cortex, although these studies did not investigate the FC [2]. A recent neuroimaging investigation suggested that OP is activated during the anticipation of severe dyspnoea caused by effort breathing [19]. Thus, increased FC between the salience network and secondary visual cortex may suggest the increased perception of dyspnoea and the prediction of its severity. The secondary visual cortex has been proposed as a “network hub” that plays an important role in diverse cognitive tasks and the dynamic coupling of functional networks [35]. The default mode network (including the superior parietal, superior frontal cortices and posterior cingulate gyrus) and the salience-processing network (ACC and AInsula) are multimodal and functional hubs [52]. Additionally, primary cortical regions, such as the primary motor and visual cortices, have been identified as hubs within single or limited functional networks [53]. To investigate the possibility that the secondary visual cortex functions as a hub, we analysed the degree and betweenness centralities using graph theoretical analysis [54]. We identified the potential of the secondary visual cortex (VO, OP, and OFusG) to serve as a hub for FC. Collectively, our study signified that effort breathing activates FCs during the sensory processing of respiration [2,50,51], primarily through network hubs within the secondary visual cortex.

Recent studies on COPD demonstrate decreased FC within the visual network, showing a positive correlation with cognitive function [55,56]. Patients with COPD exhibited the abnormal synchrony of regional spontaneous activity within the visual processing pathways, potentially linked to impaired visual and visuospatial memory [57]. Furthermore, reduced interaction between the visual network and other networks was speculated to cause cognitive impairment [56]. Our results, alongside these previous studies, highlight the role of the visual network as a key network hub of cognitive function during both acute and chronic respiratory impairment. 

### 4.4. Correlation with Clinical Score (Modified Borg Scale)

We found a positive correlation between the modified Borg scale score and values between the left AInsula and left OP FC during effort breathing. The modified Borg scale is a 12-point scoring system with ratings ranging from 0 to 10 with verbal anchors that are more convenient for comparisons between individuals and correlate well with physiological parameters during exercise testing [58,59]. The modified Borg scale is widely used to assess the intensity of dyspnoea in healthy and cardiopulmonary populations [37] and acute bronchospasms, such as COPD and asthma [58]. It has been reported that there is a strong correlation between the intensity of breathlessness, as described by the modified Borg scale, and the amount of work performed during exercise [60]. A significant negative correlation was observed between the change in the modified Borg scale and the peak expiratory flow rate (PEFR) [59], which might be affected by effort breathing. Taken together, the modified Borg scale scores may be associated with the FC values between the respiratory sensory processing and network hubs. In other words, subjective dyspnoea may be influenced by the activity of the salience network.

## 5. Limitations

Our study had several limitations. First, the relatively small sample size and inclusion of relatively young participants may limit generalisability. Second, we did not investigate the participants’ precise psychological states. Third, we focused only on resistive load-induced respiratory impairment, which cause increased workload and a sensation of mild dyspnoea. Thus, our results cannot be generalised to other qualities of respiratory impairment, such as air hunger and chest tightness. Fourth, we relied solely on the modified Borg scale to assess respiratory impairment, which may have limited our assessment. Correlating findings with other clinical scales may provide insights into the differentiation of FC linked to motor control, perception, or anticipation. Lastly, at this point, the acute model of dyspnoea is currently not applicable to chronic diseases such as asthma, COPD, obstructive sleep apnoea, and panic disorder. 

## 6. Conclusions

Our study demonstrated that mild transient effort breathing altered FC within the sensory area of respiration (salience network and PostCG) via a network hub (secondary visual cortex). One FC was positively correlated with the modified Borg scale score. This study focused on effort breathing; therefore, future research may explore the mechanisms of other respiratory impairments, such as hypercapnia, hyperpnea, and the urge to cough. Additionally, exploring the precise role of visual networks in respiratory impairment could be a valuable goal for future experiments.

## Figures and Tables

**Table 1:** Characteristics of participants at the screening visit.

Characteristics	Participants, *n* = 36
Age, years	35.0 ± 7.2
Gender, women/men, *n*	18/18
Body mass index, kg/m^2^	22.6 ± 2.8
Respiratory rates, /min	15.5 ± 3.3
Heartbeats, /min	71.0 ± 8.4
Blood pressure, mmHg	
Systolic	116.0 ± 12.7
Diastolic	68.5 ± 10.6
Saturation of percutaneous oxygen, %	97.9 ± 1.0
Hasegawa’s Dementia scale, points	29.9 ± 0.5
Lung function tests	
FVC, L	4.0 ± 0.9
%FVC predicted, %	104.3 ± 12.3
FEV_1_, L	3.4 ± 0.7
%FEV_1_ predicted, %	102.5 ± 11.3
FEV_1_/FVC ratio	0.86 ± 0.06
Blood tests	
White blood cell count, ×10^2^/µL	5.7 ± 1.1
Red blood cell count, ×10^4^/µL	475 ± 39
Haemoglobin, g/dL	14.3 ± 1.6
Haemoglobin A1c (NGSP), %	5.2 ± 0.6
Serum tests	
Thyroid-stimulating hormone, µIU/mL	1.8 ± 0.9
Blood urea nitrogen, mg/dL	11.9 ± 2.8
Creatinine, mg/dL	0.7 ± 0.1
Sodium, mEq/L	140.4 ± 1.6
Chloride, mEq/L	104.1 ± 1.9
Potassium, mEq/L	4.1 ± 0.3
Aspartate aminotransferase, U/L	22.7 ± 8.4
Alanine aminotransferase, U/L	24.7 ± 14.1
Lactate dehydrogenase, U/L	177.2 ± 32.1
Gamma-glutamyl transpeptidase, U/L	35.6 ± 45.6
Vitamin B1, ng/mL	33.9 ± 7.1

Notes: Data are expressed as mean ± standard deviation (SD) and the Japanese normal range, except for participants’ number (n). None had abnormal results in all screening tests, although five were obese (25.2, 26.1, 26.3, 26.7, and 28.4 kg/m^2^ of body mass index, respectively). Abbreviations: FEV_1_, forced expiratory volume in 1 s; FVC, forced vital capacity; NGSP, National Glycohemoglobin Standardization Program; %FEV_1_ predicted, percent predicted forced expiratory volume in 1 s of normal; %FVC predicted, percent predicted forced vital capacity of normal.

**Table 2:** Statistical cluster analysis of networks presenting increased connectivity between ROIs (effort breathing > normal respiration).

Analysis Unit (ROI-to-ROI)		Statistics	*p*-Uncorrected	*p*-FDR
		Cluster 1/325 F (3, 68) = 9.18	0.000035	0.011489
Sal AInsula l	VO	T (70) = 4.51	0.000025	0.004139
Sal AInsula l	OP r	T (70) = 4.26	0.000063	0.004326
Sal AInsula l	OFusG r	T (70) = 4.19	0.000080	0.004326
Sal ACC	OP r	T (70) = 3.92	0.000206	0.019694
Sal ACC	OFusG r	T (70) = 3.78	0.000328	0.019694
Sal ACC	VO	T (70) = 3.75	0.000362	0.019608
Sal AInsula l	OP l	T (70) = 3.52	0.000752	0.028116
Sal AInsula l	OFusG l	T (70) = 3.48	0.000862	0.028116

Abbreviations: l, left; r, right; ACC, network’s salience anterior cingulate cortex; AInsula, network’s salience anterior insular; FDR, false discovery rate; OFusG, occipital fusiform gyrus; OP, occipital pole; VO, network’s visual occipital.

**Table 3:** Clusters of voxels showing increased connectivity with salience anterior cingulate cortex (ACC) and left salience anterior insular (AInsula) in seed-to-voxel analysis.

Seed	Cluster Name	Clusters (x, y, z)	Size	Coverage	Size p-FEW	Size p-FDR	Size p-unc	Peak p-FEW	Peak p-unc
Sal. ACC	A	+06 −98 −04	858	303 voxels (35%) covering 12% of OP r	0.000011	0.000018	0.000001	0.386005	0.000017
				194 voxels (23%) covering 22% of OFusG r					
				76 voxels (9%) covering 3% of OP l					
				52 voxels (6%) covering 3% of LG l					
				31 voxels (4%) covering 3% of OFusG l					
				23 voxels (3%) covering 1% of LG r					
				18 voxels (2%) covering 1% of iLOC r					
				5 voxels (1%) covering 1% of ICC l					
				2 voxels (0%) covering 0% of Cereb 6 l					
				154 voxels (18%) covering 0% of not-labelled					
Sal. AInsular l	A	+26 −82 −04	1145	349 voxels (30%) covering 14% of OP r	0.000000	0.000001	0.000000	0.071112	0.000002
				317 voxels (28%) covering 36% of OFusG r					
				93 voxels (8%) covering 5% of iLOC r					
				12 voxels (1%) covering 2% of ICC r					
				5 voxels (0%) covering 0% of sLOC r					
				369 voxels (32%) covering 0% of non-labelled					
	B	−14 −90 +04	572	329 voxels (58%) covering 12% of OP l	0.000275	0.000291	0.000022	0.636977	0.000039
				36 voxels (6%) covering 4% of OFusG l					
				16 voxels (3%) covering 2% of ICC l					
				15 voxels (3%) covering 1% of LG l					
				176 voxels (31%) covering 0% of non-labelled					
	C	+28 −34 +72	219	185 voxels (84%) covering 6% of PostCG r	0.043271	0.031177	0.003464	0.009311	0.000000
				3 voxels (1%) covering 0% of PreCG r					
				31 voxels (14%) covering 0% of non-labelled					

Abbreviations: l, left; r, right; *p*-FEW = family-wise error corrected *p*-value; *p*-FDR = false discovery rate-corrected *p*-value; *p*-unc = uncorrected *p*-value; Cereb, cerebellum; ICC, intracalcarine cortex; iLOC, lateral occipital cortex, inferior division; LG, lingual gyrus; OFusG, occipital fusiform gyrus; OP, occipital pole; PreCG, precentral gyrus; PostCG, postcentral gyrus; sLOC, lateral occipital cortex, superior division.

**Table 4:** Identification of hubs using graph theoretical analysis (effort dyspnoea > normal respiration).

Analysis Unit	Degree Centrality			Betweenness Centrality		
	*t*-Value	*p*-Uncorrected	Rank	*t*-Value	*p*-Uncorrected	Rank
VO	3.26	0.001706	1	2.93	0.004589	2
OFusG l	2.71	0.008518	2	2.49	0.015310	5
OP l	2.26	0.026699	7	2.14	0.035570	8
OP r	2.20	0.031157	8			

Abbreviations: l, left; r, right; OFusG, occipital fusiform gyrus; OP, occipital pole; Rank, ranking among all ROIs; VO, network’s visual occipital.

## Data Availability

The datasets generated and analysed in the current study are available from a corresponding author upon reasonable request.

## Supplementary Materials

The following supporting information can be downloaded at: https://www.mdpi.com/article/10.3390/jcm13092556/s1, Figure S1: The structure of the device and the results from the preliminary study using the devices in healthy volunteers. Figure S2: Changes in modified Borg scale and vital signs during respiratory training between normal breathing (mouthpiece alone) and resistive load (device).
