# Supplementary material for: Altered Functional Connectivity during Mild Transient Respiratory Impairment Induced by a Resistive Load
Source: J Clin Med. 2024 Apr 26;13(9):2556. doi: 10.3390/jcm13092556 (PMC11084533; doi:10.3390/jcm13092556)
Supplement: Supplementary file 1 [file jcm-13-02556-s001.zip › jcm-2947402-supplementary.pdf]

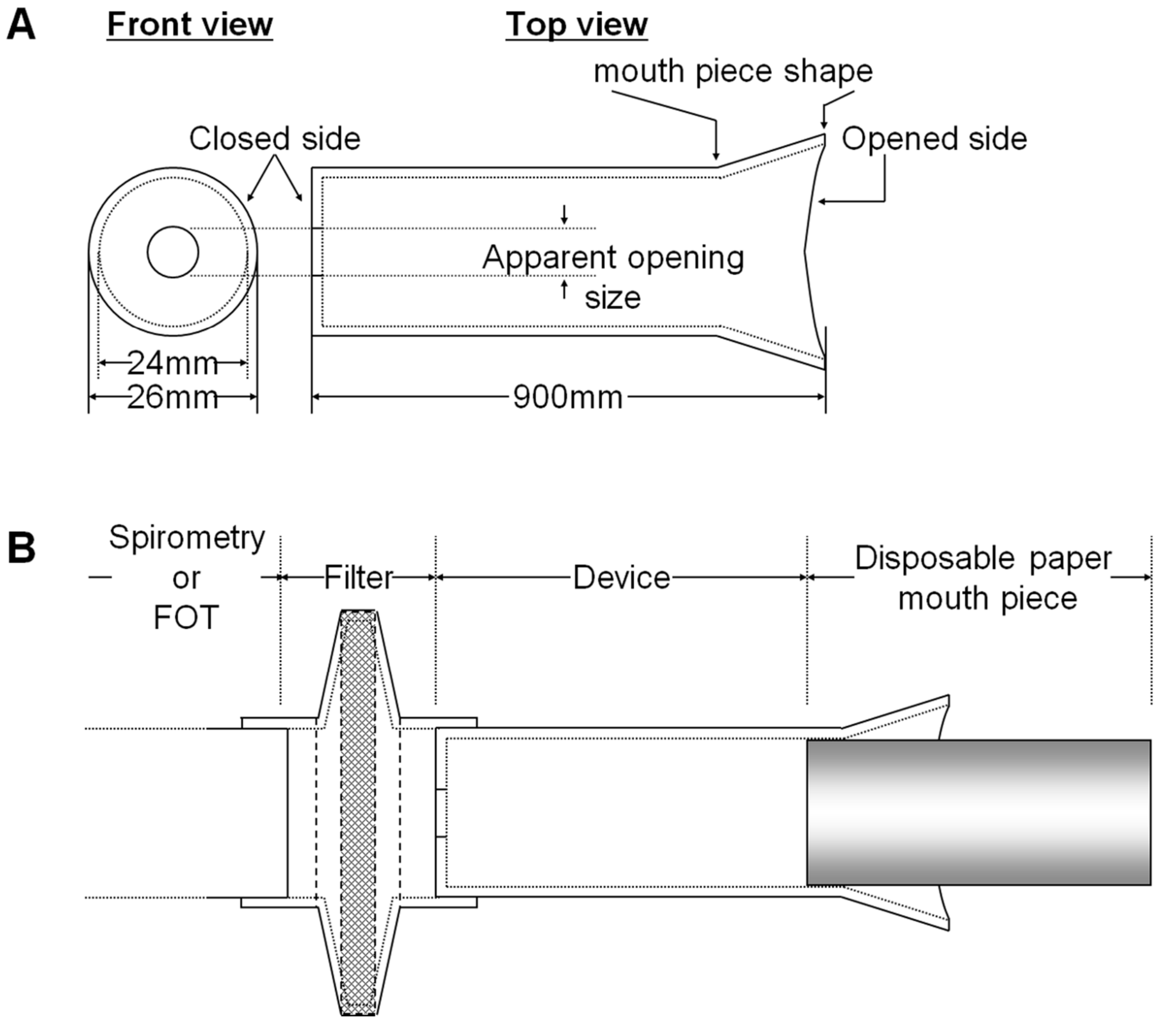


Figure S1: The structure of the device and the results from the preliminary study using the devices in healthy volunteers.

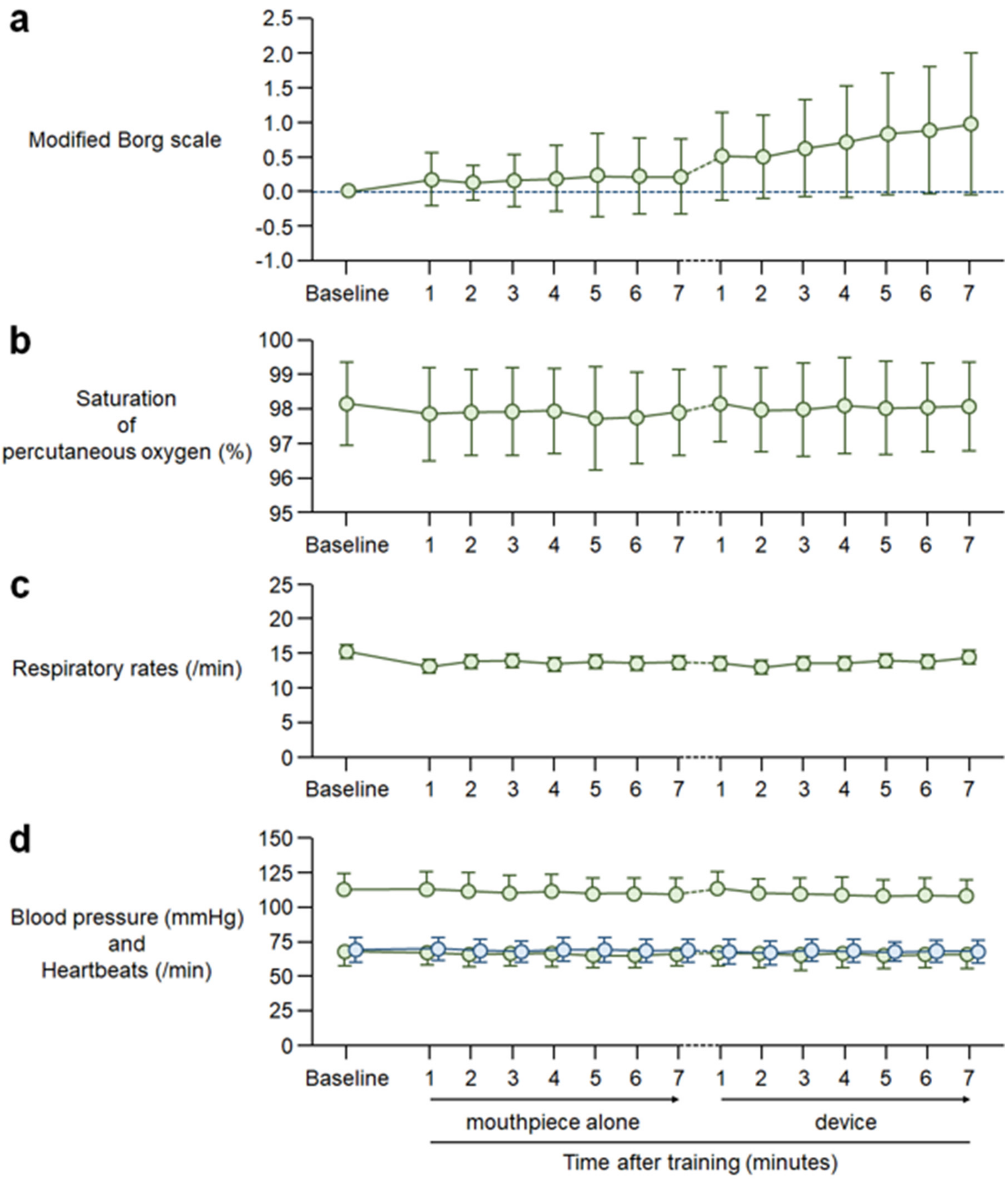


Figure S2: Changes in modified Borg scale and vital signs during respiratory training between normal breathing (mouthpiece alone) and resistive load (device).
